# Laboratory Evaluation of a Basic Recombinase Polymerase Amplification (RPA) Assay for Early Detection of *Schistosoma japonicum*

**DOI:** 10.3390/pathogens11030319

**Published:** 2022-03-04

**Authors:** Wangping Deng, Shenglin Wang, Liping Wang, Chao Lv, Yinlong Li, Ting Feng, Zhiqiang Qin, Jing Xu

**Affiliations:** National Institute of Parasitic Diseases, Chinese Center for Disease Control and Prevention (Chinese Center for Tropical Diseases Research), NHC Key Laboratory of Parasite and Vector Biology, WHO Collaborating Centre for Tropical Diseases, National Center for International Research on Tropical Diseases, Shanghai 200025, China; dengwp@nipd.chinacdc.cn (W.D.); wang20126518@163.com (S.W.); wlpnlnl@163.com (L.W.); lvchao@nipd.chinacdc.cn (C.L.); liyl@nipd.chinacdc.cn (Y.L.); fengting@nipd.chinacdc.cn (T.F.); qinzq@nipd.chinacdc.cn (Z.Q.)

**Keywords:** *Schistosoma japonicum*, RPA assay, early detection, infected mice, *Oncomelania hupensis*

## Abstract

Early detection of *Schistosoma japonicum* (*S. japonicum*) within its intermediate and definitive hosts is crucial for case finding and disease surveillance, especially in low-endemic areas. Recombinase polymerase amplification (RPA) has many advantages over traditional methods of DNA-amplification, such as polymerase chain reaction (PCR), including high sensitivity and specificity whilst being deployable in resource-poor schistosomiasis-endemic areas. Here, we evaluated the performance of a basic RPA assay targeting the 28srDNA gene fragment of *S. japonicum* (Sj28srDNA) using schistosome-infected *Oncomelania hupensis* (*O. hupensis*) and mouse models, compared to the traditional pathological method and a PCR assay. Overall *S. japonicum* infection prevalence within *O. hupensis* hosts by microscopic dissection, PCR and RPA was 9.29% (13/140), 32.14% (45/140) and 51.43% (72/140), respectively, presenting significant differences statistically (χ^2^ = 58.31, *p* < 0.001). It was noteworthy that infection prevalence by PCR and RPA performed was 34.44% (31/90) and 53.33% (48/90) in snails within 6 weeks post-infection, while the dissection method detected all samples as negatives. In addition, the basic RPA assay presented positive results from the fourth week post-infection and third day post-infection when detecting fecal DNA and serum DNA, respectively, which were extracted from a pooled sample from mice infected with 20 *S. japonicum* cercariae. This study suggests that the RPA assay has high potential for early detection of *S. japonicum* infection within its intermediate and definitive hosts.

## 1. Introduction

Schistosomiasis is a zoonotic parasitic disease prevalent throughout 78 countries, afflicting more than 250 million people worldwide, while schistosomiasis japonica caused by *Schistosoma japonicum* (*S. japonicum)* localizes in China, the Philippines, and a small pocket of Indonesia in Asia [1,2]. Nowadays, remarkable achievements in schistosomiasis japonica control have been gained in these endemic countries through several decades of endeavor, especially in China [3,4]; 5 of 12 previous endemic provinces in China have been pioneers in schistosomiasis elimination. In 2020, only 3 cases (3 out of 273, 312) were found to be etiologically positive nationwide [5]. The elimination of schistosomiasis has been placed on the agenda of the national strategic plan of Healthy China 2030 [6]. *S. japonicum* has a complex life cycle that requires oncomelanid snails as intermediate hosts and mammalian animals as definitive hosts. Briefly, mature adult worms reside in the mesenteric veins of definitive hosts, where female worms lay eggs; then, partial eggs trapped in the liver, cause inflammatory immune responses (including granulomas) that result in intestinal disease, whereas the other eggs are discharged into environments via feces and hatch to miracidia in freshwater to penetrate snail hosts. Schistosomes develop in snails through asexual reproduction and release thousands of cercariae into the water body. Humans and other mammals are infected when they contact infectious water containing *S. japonicum* cercariae, which actively penetrate mammalian skin and invade the body. Thus, detection of *S. japonicum* at different life stages in intermediate or definitive hosts can provide vital information for disease surveillance and control [7,8]. However, it is extremely crucial to apply optimal detection tools that adapt to different stages of schistosomiasis control. Parasitological tests, such as the Kato-Katz method (KK) and miracidia hatching technique (MHT), and immunological technologies [9,10], mainly including indirect hemagglutination assay (IHA), the enzyme-linked immunosorbent assay (ELISA), as well as some rapid diagnostic tests (RDTs), have been developed and applied for the case finding in national control programs for diagnosing schistosomiasis [11,12]. Yet, the limitations of low sensitivity for parasitological methods and incapability of distinguishing past and current infections for serological techniques are more apparent with the decrease in prevalence and infection intensity of schistosomiasis in human beings and livestock [13,14,15,16,17,18]. Meanwhile, the detection of infected snails by traditional microscopic dissection is likely to miss infection in the early stage, particularly in low-transmission settings. Thus, an optimal approach with extreme sensitivity and specificity is urgently needed for the detection of asymptomatic cases or light infections, as well as disease surveillance to verify transmission interruption or elimination.

With the development of molecular diagnostic technology, nucleic acid detection methods including conventional polymerase chain reaction (PCR) [19], nested PCR [20], real-time PCR [21,22,23], digital PCR [24], and so on [25], presenting high detection specificity and sensitivity [26,27], provide a new idea for the diagnosis of schistosomiasis. However, these PCR-based techniques require expensive equipment and skilled personnel, which are rarely available in schistosomiasis endemic areas featured by poverty and scarce of resources [28]. Recently, alternative and portable DNA amplification technologies [29] have been developed, such as loop-mediated isothermal amplification (LAMP) [30,31,32] and recombinase polymerase amplification (RPA) [33,34,35,36,37]. It is reported that LAMP can be used for molecular xenomonitoring of infected snail which may avoid missing early infections [32,38]. However, RPA is regarded as a novel isothermal amplification technology with more advantages than LAMP such as rapidity, ease of use, and suitability for field use in point-of-care settings [39]. It is based on the nucleic acid replication mechanism of T4 bacteriophage, and the results can be interpreted within 20 min at a wide temperature range (25–45 °C) without any specialized or expensive equipment [40].

In previous report, the visual detection method LFD-RPA (RPA combined with lateral flow dipstick (LFD) and the real time RPA (RT-RPA) target SjR2 gene have been developed for detection of *S. japonicum* infection in humans and goats using stool and plasma samples, respectively, which presented with high sensitivity and high specificity [36,41]. However, there has been no report on the detection of infected snails by the RPA method, particularly in the early stage of infected snails. In our previous work, we developed a basic RPA assay targeting Sj28srDNA, presenting extreme specificity without cross reaction with other parasites and high sensitivity of capable detecting 100 fg of *S. japonicum* DNA in 20 min at 39 °C [42]. In this study, to explore its potential for identifying *S. japonicum* infection in the early stages within its intermediate and definitive hosts, we assessed the analytical performance of the basic RPA assay in the infection process through infected mouse and snail models in the laboratory, compared with the microscopic dissection method and a PCR assay with a single-blind manner.

## 2. Results

### 2.1. Detection of S. japonicum-Infected Snails by Microscopy, PCR and RPA

**Figure 1:**
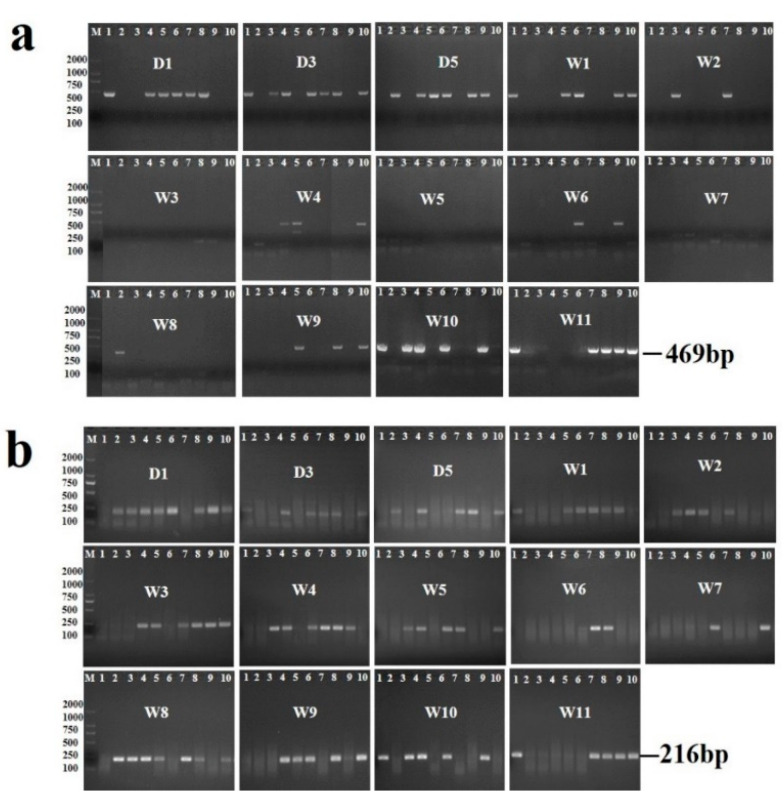
The results of the PCR assay (**a**) and RPA assay (**b**) for detecting *S. japonicum* in snails at different time points post-infection. The snail DNA was from 14 time points post-infection; ten snail DNA samples were tested at each time point, and each lane showed the result of one DNA sample of a snail. M represented the DNA size marker. D1, D3, D5, W1, W2, W3, W4, W5, W6, W7, W8, W9, W10 and W11 represented the time point of snail collection post-infection. Note: D is the abbreviation of Day, W is the abbreviation of Week. The amplicon size by PCR is 469 bp, while this is 216 bp by the RPA assay.

In total, 140 experimentally infected snails were collected at 14 time points post-infection; which were all tested by the microscopic dissection method, PCR and RPA assay in parallel. The positive rates of snails at each time point post-infection were listed in Table 1, while the electrophoresis images of the PCR and RPA amplified products are shown in Figure 1. The overall positive rates of snails determined by microscopic dissection method, PCR and RPA assay were 9.29% (13/140), 32.14% (45/140) and 51.43% (72/140) respectively, showing significant difference among them (*χ*^2^ = 58.31, *p* < 0.001). In detail, totally 13 snails were found positive by the microscopic dissection method all after 6 weeks post-infection while the left 127 snails were negative. The positive rates of schistosoma infection in snails determined by microscopic dissection method during 7th–11th weeks ranged from 10–40%. Specifically, among the 13 positives determined by the microscopic dissection method, all were tested as positive results by RPA assay while 9 of them were positive by PCR assay (Table 2). The positive rates of PCR and RPA at each time point post-infection varied from 0 to 70% and 20% to 80%, respectively. 

Taking the microscopic dissection method as reference method, the performance of the PCR assay and RPA assay were assessed and presented in Table 2. The sensitivity and specificity of PCR were 69.23 % (95% *CI*: 38.88–89.64%) and 71.65% (95% *CI*: 62.86–79.11%), respectively, while RPA performed 100% (95% *CI*: 71.66–100%) of sensitivity and 53.54% (95% *CI*: 44.51–62.36%) of specificity.

The average snail positive rates by PCR and RPA within 6 weeks were 34.44% (31/90) and 53.33% (48/90), respectively, with a significant difference detected (*χ*^2^ = 6.52, *p* = 0.011). In addition, the positive rates after 6 weeks post infection determined by microscopic examination, PCR and RPA were 26.00% (13/50), 28.00% (14/50) and 48.00% (24/140), respectively, showing significant difference among them (*χ*^2^ = 6.60, *p* = 0.037). This is shown in Table 3. 

### 2.2. Detection of S. japonicum-Infected Mice by PCR and RPA

#### 2.2.1. Confirmation of Worm Burden in Infected Mice Models

Schistosome worms perfused from each dissected mouse after 6 weeks of exposure were counted. The mean worm burden of mice infected with 5 (group A), 10 (group B) or 20 (group C) cercariae was 3.1 ± 1.2, 6.5 ± 2.2 and 12.4 ± 4.8 worms per mouse, respectively, indicating that three groups of the infected mice model with gradient infection intensity were successfully established.

#### 2.2.2. Examination of Fecal DNA

**Figure 2:**
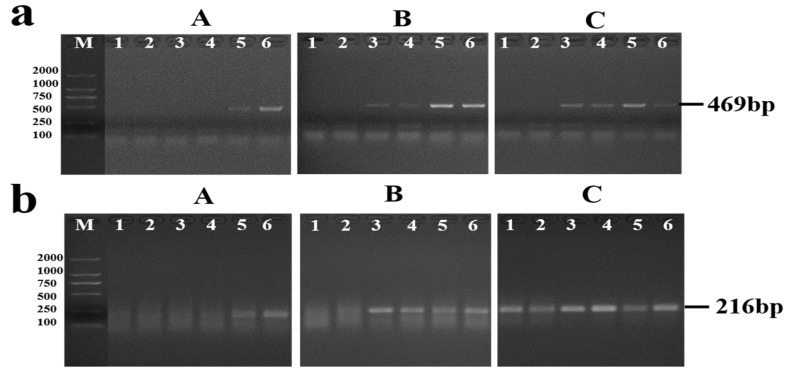
Examination of fecal DNA by PCR assay (**a**) and RPA assay (**b**), A: group A (5 cercariae), B: group B (10 cercariae), C: group C (20 cercariae), For (**a**,**b**), lane1 and lane 2 represented the parallel results of the fourth week post infection, Lane 3 and lane 4 represented the results of the fifth week post infection, lane 5 and lane 6 represented the results of the sixth week post infection.

The results of the PCR and RPA assay were all negative when used to detect the DNA samples extracted from pooled feces in all mice group from the third day to the third week post-infection. For PCR, positives were initially presented since the sixth week in group A, and the fifth week both in group B and C (Figure 2a). RPA initially presented positive results from the sixth week post-infection in group A, the fifth week post-infection in group B, and the fourth week post-infection in group C (Figure 2b). 

#### 2.2.3. Examination of Serum DNA

**Figure 3:**
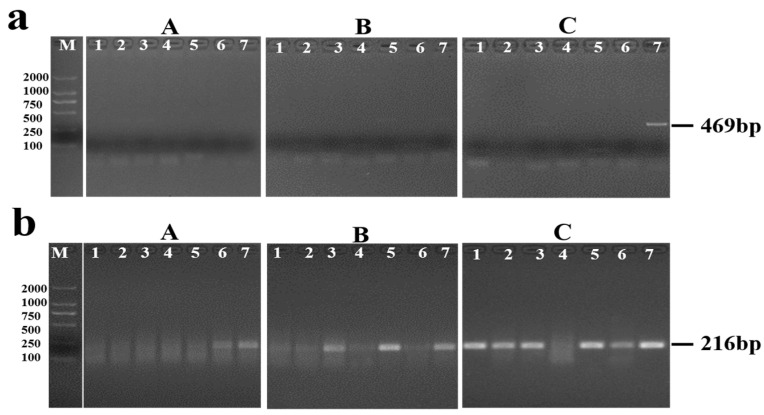
Examination of serum DNA by PCR assay (**a**) and RPA assay (**b**). A: group A (5 cercariae), B: group B (10 cercariae), C: group C (20 cercariae). For (**a**,**b**), lane 1–7 represented the results of the third day, first week, second week, third week, fourth week, fifth week and sixth week post-infection, respectively.

When detecting the DNA extracted from pooled sera of each mice group by PCR assay, positives were only detected when the sera samples collected at the fourth and sixth week post-infection in group B, and at the second and sixth week post-infection in group C (Figure 3a). For RPA assay, the positive results for detecting *S. japonicum* were presented at the fifth and sixth week post infection in group A, at the second, fourth and sixth week post infection in group B, and at the third day, the first, second, fourth, fifth and sixth week post-infection in group C (Figure 3b). 

## 3. Discussion

China used to have the highest disease burden of schistosomiasis japonica worldwide. The disease was endemic in 12 provinces along the Yangtze River with over 2100 years of history. It was estimated that about 11.6 million people were infected by *S. japonicum* in 1950s. Through seven decades of efforts, the morbidity and transmission of schistosomiasis were well under control and a new goal to eliminate schistosomiasis by 2030 nationwide was put forward [6]. To fulfill the elimination of schistosomiasis, extreme sensitivity and specificity tools were needed for case finding and disease surveillance under the current situation with very low prevalence and infection intensity of schistosomiasis in human beings and snails. Although several molecular techniques based on PCR methods have been established in prior studies and presented great advantages with higher sensitivity and specificity to detect *S. japonicum* infections over traditional methods, the shortcomings of dependence on expensive equipment, long reaction time to get results or low detection rate to early or light infections etc. restrict their application in point-of-care setting. Being a potential technique implemented in field settings, RPA can operate at 37–42 °C, amplify as low as 1–10 copies of target DNA and get results in less than 20 min. In addition, it has been successfully integrated with different detection strategies, making it more user-friendly. In this study, we explored preliminarily the performance of the basic RPA assay developed in our previous study for early detection of *S. japonicum* based on *S. japonicum* infection model in snails and mice.

Firstly, the performance of RPA was assessed through examining 140 experimental snails at different time points post *S. japonicum* infection, in parallel microscopic examination and the PCR assay were conducted with single-blind manner. The results showed that all 13 positive snails identified by microscopy were confirmed positive by RPA, while only 9 of them were positive determined by PCR. Taking microscopic dissection method as the reference test, RPA assay performed higher sensitivity than that of PCR assay (100% versus 69.23%). Furthermore, we noticed that RPA presented advantages when identifying early infection in snails. In our studies, the positive rates of PCR assay and RPA assay were 34.44% and 53.33% respectively when detecting the snails within 6 weeks post infection, while no positive snail was observed by microscopic examination. This may because it usually takes about 60 days for schistosome to develop into cercariae in snails, and it is difficult to observe mother sporocyst in the early infection stage by microscopic examination, while PCR and RPA can detect positive snails once schistosome nucleic acid exist in the snails in the early stage. All the results mentioned above suggested that the RPA performed great potentials as a surveillance tool to identify risk foci or hot spots of schistosomiasis. However, we also noticed that the results of RPA and PCR were not always consistent. For example, there were 9 snails which were positive determined by PCR but negative by RPA and microscopy in the early stage post-infection, and 32 snails were identified positive by RPA, but negative by PCR and microscopy dissection method. The former may largely be explained by the operational error when preparing reaction mixture or false positive reaction of PCR, while the later may be attributed to the higher sensitivity of RPA over PCR which had been proved in our previous work that the limit of detection of basic RPA assay was ten-fold lower than PCR [42]. Further evaluation through increasing the sample size of each time points may elucidate the reason of results difference between PCR and RPA. 

Moreover, we explored preliminarily the possibility of PRA assay for detecting the early infection of *S. japonicum* in its definitive hosts using mice models. When detecting fecal samples, the RPA and PCR assay presented positive results since the fourth week post-infection and the fifth week post-infection separately in the mice group infected with 20 cercariae. But no positive was detected by RPA assay in fecal samples within fourth week post-infection in other two mice groups, which featured a low-intensity infection. Similarly, the detection efficiency of RPA and PCR assay was influenced by the infection intensity of schistosomes when detecting sera samples collected from mice model. As previous studies showed the cell-free circulating schistosome DNA in serum mainly derived from the dead worms during migration or the epidermal tissue cells or exosomes of worms secreted into the host’s circulation system, or released by the disintegration of eggs, we supposed the performance of RPA and PCR for detecting sera DNA may influenced by the amount of the cell-free nucleic acid in the circulating system and the quality of DNA extraction [26,43]. Based on the positive results of RPA appeared earliest at third day post-infection in the mice groups infected with 20 cercariae, RPA presented higher early detection potential for *S. japonicum* infected mice and further verification should be conducted. 

Being an explorative study, there were still some limitations. First, the infected snail model in our study was established by pooling method, it couldn’t ensure each snail infected with miracidia, thus we particularly compared the detection rates among microscopic examination, RPA and PCR. Second, only pooling fecal and sera samples at each time point were collected and detected by RPA and PCR as large amount of samples needed to extract enough DNA but we only have a small number of mice in each group and could not get enough samples from individual mouse at each time point by continuous collection. Further evaluation should be conducted through increasing sample size to comprehensively evaluate the efficiency of RPA using samples collected from individual mouse. Finally, being pivotal to any molecular method, the procedure and quality of DNA extraction may impact the performance of RPA and its application for field use. Simplifying the DNA extraction procedures with high quality in field stings is a big challenge to be solved urgently.

## 4. Materials and Methods

### 4.1. Establishment and Microscopic Screening of S. japonicum-Infected O. hupensis

Laboratory-reared *O. hupensis* snails were mixed with miracidia of *S. japnonicum* provided by Jiangsu Institute of Parasitic Diseases, which were mixed at the ratio of 1:20 in dechlorinated water in a 10 cm culture dish. Snails were exposed to light at 25 °C for 2 h to encourage miracidia invasion of snail tissues. Ten snails were selected at each time point of the first, third and fifth day, as well as at the first to eleventh week post-infection setting one week as the time interval. The infection status of each snail was tested by microscopic examination. Successful infection and development were confirmed if mother sporocysts, daughter sporocysts or cercariae of *S. japnonicum* were observed in the snails. Then, the soft tissue of snails was collected in tubes after removing the crushed snail shell and stored in a −20 °C refrigerator for subsequent DNA extraction.

### 4.2. Establishment of S. japonicum-Infected Mouse Models and Subsequent Fecal and Serum Sampling

*S. japonicum* cercariae were released from the experimentally infected snails (*O. hupensis*). Thirty-three female BALB/c mice aged from 6 to 8 weeks, were randomly divided into 3 groups. Each mouse in group A, B, and C was infected with 5, 10 and 20 *S. japonicum* cercariae, respectively, through covering the shaved abdominal skin using Cercariae-carrying slide. Blood samples of each mouse were collected from tail vein at the third day and from first to sixth week post-infection, respectively. Mixed fecal samples of each group were collected at the same time points as blood sample collection respectively. Serum samples were separated by centrifugation (3500× *g* for 10 min) after storage at room temperature for 2 h. Then, 50 μL of 10 serum samples at the same time point in each group were mixed. All the samples were kept frozen at −20 °C until use. At the sixth week post-infection, all mice were dissected and perfused to count the adult worm burden of each mouse.

### 4.3. DNA Extraction from Snail, Feces and Sera Samples 

We optimized the method of DNA extraction from feces and sera through the spiked-in samples (adding genomic DNA in sera, egg in feces), and using the verified-infected snail for control of DNA extraction from snails. The optimal kit and protocol for DNA extraction from snails, feces and sera in our lab were confirmed. In this study, snail tissue DNA was extracted using the DNAeasy Blood and Tissue Kit (Qiagen, Hilden, German) Fecal DNA was extracted using the QIAamp fast DNA stool mini kit (Qiagen, Hilden, German) with slight modification. Briefly, 200 mg of feces for each sample were mixed in 500 μL of Inhibit EX Buffer and then grinded by a tissue grinder. The extraction procedure was performed according to the manufacturer’s instructions. The E.Z.N.A.^®^ Circulating DNA Kit (Omega Bio-tek, Inc., Norcross, GA, USA) was used to extract the serum DNA as instructed by the manufacturer, beginning with dissolving 200 μL of mixed mice sera in 200 μL lysis buffer provided by the kit. All the DNA samples were eluted using 50 μL distilled water which does not influence the RPA and PCR reaction. In addition, the DNA concentrations were determined by Nano Drop 2000, and were stored at −20 °C to avoid degradation until analyzed.

### 4.4. Detection and Amplific Ation of S. japonicum DNA by PCR and RPA

All DNA samples were tested by the PCR assay and RPA assay. The PCR primer refers to the sequence published by Chen et al. [19]. The PCR reaction was performed in a total reaction volume of 50 μL containing: 25 μL of PCR master mix (2xHieff Tm PCR mix, Yeasen, Shanghai), 2 μL of each primer, 1 μL of snail DNA, fecal DNA or serum DNA for each reaction(Adult worm genomic DNA or verified infected snail DNA were used as the positive control for each batch of reaction, and ddH_2_O or verified negative snail DNA or normal mouse DNA were used as the negative control), as well as 20μL of ddH_2_O. The thermal cycling condition for PCR was as follows: 95 °C for 5 min (initial denaturation), followed by 39 cycles at 95 °C for 20 secs (denaturation), 56 °C for 60 s (annealing), 72 °C for 40 s (extension) and a final extension at 72 °C for 5 min. The PCR products were analyzed by electrophoresis using 2.0% agarose gel, and the target PCR amplification product size was 469 bp.

The basic RPA primers designed in our previous work, are shown in Table 4. The target sequence was the gene fragment of Sj28srDNA (GenBank No: Z46504.4). The RPA reaction were performed using the TwistAmp basic kit (TwistDX, Cambridge, UK) in 50 μL reaction volume. The reaction mixture contained 29.5 μL rehydration buffer, 12.2 μL of ddH_2_O, 2.4 μL of each primer, and 1 μL of snail DNA, fecal DNA or serum DNA for each reaction (Adult worm genomic DNA or verified infected snail DNA were used as the positive control for each batch of the reaction, and ddH_2_O or verified negative snail DNA or normal mouse DNA was used as the negative control.). Then, the mixture was added to the lyophilized RPA pellet. The reactions were initiated by the addition of 2.5 μL (280 mM) of magnesium acetate. The tubes were then incubated at 39 °C in a water bath for 20 min. In the last step, the RPA products were analyzed by electrophoresis using 2% agarose gel. Positives were determined if a 216 bp band was presented in the gel. 

### 4.5. Ethical Statement 

All animal experiments were carried out according to the Recommendations and the Guide for the Care and Use of Laboratory Animals of the Ministry of Science and Technology of the People’s Republic of China, and all efforts were made to minimize suffering. The animal experiment was approved by the Ethics Committee of the Institute for Parasitic Disease Control and Prevention, China Center for Disease Control and Prevention, with the approval number of IPD-2019-18.

### 4.6. Data Management and Statistics 

The data were entered in Microsoft Excel 2013, the positive rate of snail samples was calculated by dividing the number of positive samples determined by the tested method by the total number of samples. All the analyses were performed by the R software (v. 3.6.1). The “vcd” package was used to perform a Pearson Chi-square test to compare the difference in the positive results of snail samples by microscopic examination, PCR and RPA assay. A *p*-value of ≤0.05 was considered significant. The “report ROC” package was used to calculate the sensitivity and specificity with 95% confidence intervals

## 5. Conclusions

In this study, the performance of a basic RPA assay for detecting early infection of schistosomes was evaluated by comparing with a traditional pathological test and PCR assay in the laboratory. The laboratory-based evaluation indicated that the established RPA assay presented a higher positive rate than PCR and microscopic examination when detecting infected snails and mice, showing its advantages of detecting early infection of *S. japonicum*. Further studies should be conducted to improve the performance of RPA and systematically evaluate its detection efficiency for early or light infection of *S. japonicum*, thus providing a sensitive and rapid tool for case finding and diseases surveillance.

## Figures and Tables

**Table 1:** Infection status of snails confirmed by microscopic dissection method, PCR and RPA assay.

Time Point *	Number of Snails Tested	Number of Positive Snails (Positive rate)		
		Microscopy	PCR	RPA
D1	10	0 (0%)	6 (60%)	8 (80%)
D3	10	0 (0%)	7 (70%)	6 (60%)
D5	10	0 (0%)	6 (60%)	5 (50%)
W1	10	0 (0%)	5 (50%)	6 (60%)
W2	10	0 (0%)	2 (20%)	4 (40%)
W3	10	0 (0%)	0 (0%)	6 (60%)
W4	10	0 (0%)	3 (30%)	6 (60%)
W5	10	0 (0%)	0 (0%)	5 (50%)
W6	10	0 (0%)	2 (20%)	2 (20%)
W7	10	1(10%)	0 (0%)	2 (20%)
W8	10	3 (30%)	1(10%)	7 (70%)
W9	10	2 (20%)	3 (30%)	5 (50%)
W10	10	4 (40%)	5 (50%)	5 (50%)
W11	10	3 (30%)	5 (50%)	5 (50%)
Total	140	13 (9.29%)	45 (32.14%)	72 (51.43%)

* D is the abbreviation of Day, W is the abbreviation of Week.

**Table 2:** Performance of the PCR assay and RPA assay taking microscopic dissection method as the reference test.

Assay	Number of True Positive	Number of True Negative	Sensitivity% (95% *CI* *)	Specificity% (95% *CI* *)
PCR	9	91	69.23 (38.88–89.64)	71.65 (62.86–79.11)
RPA	13	68	100 (71.66–100)	53.54 (44.51–62.36)

* 95% *CI*: 95% confidence intervals.

**Table 3:** Comparison of the positive rates determined by microscopic examination, RPA and PCR assay for detecting snails within and after six weeks post-infection of *S. japonicum*.

Infection Stage	Positive Rate (%, *n*/N)			*χ* ^2^	*p*
	Microscopy	PCR	RPA		
Within 6 weeks	0 (0/90)	34.44 (31/90)	53.33 (48/90)	6.52 *	0.011
After 6 weeks	26.00 (13/50)	28.00 (14/50)	48.00 (24/50)	6.60	0.037
Total	9.29 (13/140)	32.14 (45/140)	51.43 (72/140)	58.31	<0.001

* This value is calculated by data between RPA and PCR, as no infection was found by microscopy group.

**Table 4:** Primer sequences of PCR assay and RPA assay.

Assay	Primers (5′-3′)	Product Size (bp)	Reference
PCR	FP (851–873): TTCCGATAACGAACGAGAC	469	[42]
	RP (1299–1319): AGCGATAAAGCCACTACAAC		
RPA	FP (851–881): CTTCGGTTAGTCTGCTGATGGCTTGTTTATG	216	[19]
	RP(1066–1034) CCTTAGTTCGGACTGAAAGCCGAACCTTTACC		

## Data Availability

The data presented in this study are available in the article.
